# Recent advances on *Dirofilaria repens* in dogs and humans in Europe

**DOI:** 10.1186/s13071-018-3205-x

**Published:** 2018-12-19

**Authors:** Gioia Capelli, Claudio Genchi, Gad Baneth, Patrick Bourdeau, Emanuele Brianti, Luís Cardoso, Patrizia Danesi, Hans-Peter Fuehrer, Alessio Giannelli, Angela Monica Ionică, Carla Maia, David Modrý, Fabrizio Montarsi, Jürgen Krücken, Elias Papadopoulos, Dušan Petrić, Martin Pfeffer, Sara Savić, Domenico Otranto, Sven Poppert, Cornelia Silaghi

**Affiliations:** 1 Laboratory of Parasitology, National reference centre/OIE collaborating centre for diseases at the animal-human interface, Istituto Zooprofilattico Sperimentale delle Venezie, Legnaro, Italy; 2 Department of Veterinary Medicine, Università degli Studi di Milano, Milan, Italy; 3 Koret School of Veterinary Medicine, The Hebrew University, Rehovot, Israel; 4 Veterinary School of Nantes ONIRIS, University of Nantes, LUNAM, Nantes, France; 5 Department of Veterinary Science, Università degli Studi di Messina, Messina, Italy; 6 Department of Veterinary Sciences, School of Agrarian and Veterinary Sciences, University of Trás-os-Montes e Alto Douro, Vila Real, Portugal; 7 Institute of Parasitology, University of Veterinary Medicine, Vienna, Austria; 8 Department of Veterinary Medicine, University of Bari, Valenzano, Italy; 9 Department of Parasitology and Parasitic Diseases, University of Agricultural Sciences and Veterinary Medicine Cluj-Napoca, Cluj-Napoca, Romania; 10 Global Health and Tropical Medicine (GHTM), Instituto de Higiene e Medicina Tropical (IHMT), Universidade Nova de Lisboa (UNL), Lisboa, Portugal; 11 Department of Pathology and Parasitology, University of Veterinary and Pharmaceutical Sciences, Brno, Czech Republic; 12 Biology Centre, Institute of Parasitology, Czech Academy of Sciences, České Budějovice, Czech Republic; 13 Institute for Parasitology and Tropical Veterinary Medicine, Freie Universität Berlin, Berlin, Germany; 14 Laboratory of Parasitology and Parasitic Diseases, Faculty of Veterinary Sciences, Aristotle University of Thessaloniki, Thessaloniki, Greece; 15 Laboratory for medical and veterinary entomology, Faculty of Agriculture, University of Novi Sad, Novi Sad, Serbia; 16 Institute of Animal Hygiene and Veterinary Public Health, Veterinary Faculty, University of Leipzig, Leipzig, Germany; 17 Scientific Veterinary Institute “Novi Sad”, Novi Sad, Serbia; 18 Swiss Tropical and Public Health Institute, Basel, Switzerland; 19 University Basel, Basel, Switzerland; 20 National Centre of Vector Entomology, University of Zurich, Zurich, Switzerland; 21 Institute of Infectology, Friedrich-Loeffler-Institute, Isle of Riems, Greifswald, Germany

**Keywords:** *Dirofilaria repens*, Vector-borne infections, Mosquitoes, Zoonosis, Emergent parasite, One Health

## Abstract

*Dirofilaria repens* is a nematode affecting domestic and wild canids, transmitted by several species of mosquitoes. It usually causes a non-pathogenic subcutaneous infection in dogs and is the principal agent of human dirofilariosis in the Old World. In the last decades, *D. repens* has increased in prevalence in areas where it has already been reported and its distribution range has expanded into new areas of Europe, representing a paradigmatic example of an emergent pathogen. Despite its emergence and zoonotic impact, *D. repens* has received less attention by scientists compared to *Dirofilaria immitis*. In this review we report the recent advances of *D. repens* infection in dogs and humans, and transmission by vectors, and discuss possible factors that influence the spread and increase of this zoonotic parasite in Europe. There is evidence that *D. repens* has spread faster than *D. immitis* from the endemic areas of southern Europe to northern Europe. Climate change affecting mosquito vectors and the facilitation of pet travel seem to have contributed to this expansion; however, in the authors’ opinion, the major factor is likely the rate of undiagnosed dogs continuing to perpetuate the life-cycle of *D. repens*. Many infected dogs remain undetected due to the subclinical nature of the disease, the lack of rapid and reliable diagnostic tools and the poor knowledge and still low awareness of *D. repens* in non-endemic areas. Improved diagnostic tools are warranted to bring *D. repens* diagnosis to the state of *D. immitis* diagnosis, as well as improved screening of imported dogs and promotion of preventative measures among veterinarians and dog owners. For vector-borne diseases involving pets, veterinarians play a significant role in prevention and should be more aware of their responsibility in reducing the impact of the zoonotic agents. In addition, they should enhance multisectorial collaboration with medical entomologists and the public health experts, under the concept and the actions of One Health-One Medicine.

**Electronic supplementary material:**

The online version of this article (10.1186/s13071-018-3205-x) contains supplementary material, which is available to authorized users.

## Background

Amongst mosquito-transmitted nematodes with a zoonotic potential, *Dirofilaria repens* and *Dirofilaria immitis* (Spirurida: Onchocercidae) play significant roles from a public health perspective. *Dirofilaria immitis* causes a severe disease (heartworm disease) in dogs and other carnivores and occasionally infects humans, while *D. repens* usually causes a non-pathogenic subcutaneous infection in dogs and it is the principal agent of human dirofilariosis in the Old World [1].

*Dirofilaria repens* Railliet & Henry, 1911 (subgenus *Nochtiella*) is endemic in many countries of the Old World [2] and affects domestic and wild canids [3]. In these hosts, the adult worms are usually beneath the skin, in the subcutaneous tissues, whereas microfilariae circulate in the blood stream and are ingested by several species of competent mosquito vectors during their blood-feeding.

Microfilaremic dogs are the most important reservoir of infection, with wild canids and domestic and wild felids rarely positive for circulating microfilariae [3, 4]. In humans the parasite does not usually reach the adult stage and remains confined to an immature form. It may cause a larva migrans syndrome and form subcutaneous nodules. The worm often reaches the ocular region and occasionally other organs, such as the lungs [1, 5–7].

In the last decades, *D. repens* has increased its prevalence in areas where it has already been reported and its distribution range has expanded into new areas of Europe, with new clinical cases in both dogs and humans increasingly reported [7–11]. Thus, *D. repens* can be considered as a paradigmatic example of an emergent pathogen.

Despite its emergence and zoonotic impact, *D. repens* has received less attention by scientists compared to *D. immitis*. A thematic search in PubMed (accessed 1st May 2018) of papers focused on *D. repens* only (*repens* and NOT *immitis* in title/abstract and *vice versa*), resulted in approximately one fifth of the number of publications compared to *D. immitis* (i.e. 345 *vs* 1817). Consequently, many aspects of *D. repens* infection and epidemiology are still poorly known, for example its pathogenicity, geographical distribution, therapy and genomics.

In this paper we review the recent advances of *D. repens* infection in dogs, humans and transmission by vectors, and discuss possible factors that influence the spread and increase of the prevalence of this zoonotic parasite in Europe.

## History of *Dirofilaria repens* in dogs and humans

The first observation of *D. repens* was likely reported in a human being in 1566 by Amato Lusitano, a Portuguese medical doctor, who stated in his *Curationum Medicinalium Centuriae* “*puella trima … per oculi internam partem, quam angulum magnum appellamus, a jumbrici cuius dam caput appere coepis…*” (in a 3-year-old girl, in the area we call big angle of the eye, suddenly it started to appear the tip of one worm which sometimes is sited in the eye making its opacity) [12]. Between 1864–1879, three reports were published in Europe (Italy and Hungary) on subcutaneous and ocular human infections (reviewed in [13]), before Addario’s paper on *Filaria conjunctivae* [14], later considered synonymous with *D. repens* [15]. Ercolani [16] demonstrated that when no worms are found in the heart of microfilaremic dogs, they are usually present in subcutaneous connective or in other sites of the body, suggesting that two species of *Dirofilaria* were involved in canine filarial infections. Filarial larvae of *D. repens* collected from dogs captured in the Roma area (Italy) as well as in mosquitos were most likely described by Fulleborn [17], although at that time there was a notable uncertainty in the classification of filarial worms obtained both from the subcutaneous tissues of dogs and from ocular localization in humans. For instance, “fully developed” filariae in subcutaneous tissue of microfilaremic dogs were misdiagnosed as *Filaria immitis* in Pisa and in Milan [18]. In the first experiments to demonstrate the ability of mosquitoes to transmit parasites throughout their puncture, it is probable that *D. repens* larvae were used and not *D. immitis* as erroneously stated, as the adult worm was found in subcutaneous tissues [19]. *Dirofilaria repens* Railliet & Henry, 1911 was first described and named in 1911 on the basis of specimens sent by Bonvicini, a clinician professor of Bologna [20]. Some years later, the L1-L3 development of the parasite in the mosquito intermediate host was elucidated [21]. As far as the clinical presentation of the infection is concerned, dermatitis by *D. repens* was reported in dogs [22–24] although no clear etiological evidence was provided.

### Geographical distribution of *Dirofilaria repens* in dogs, humans and mosquitoes

**Fig. 1:**
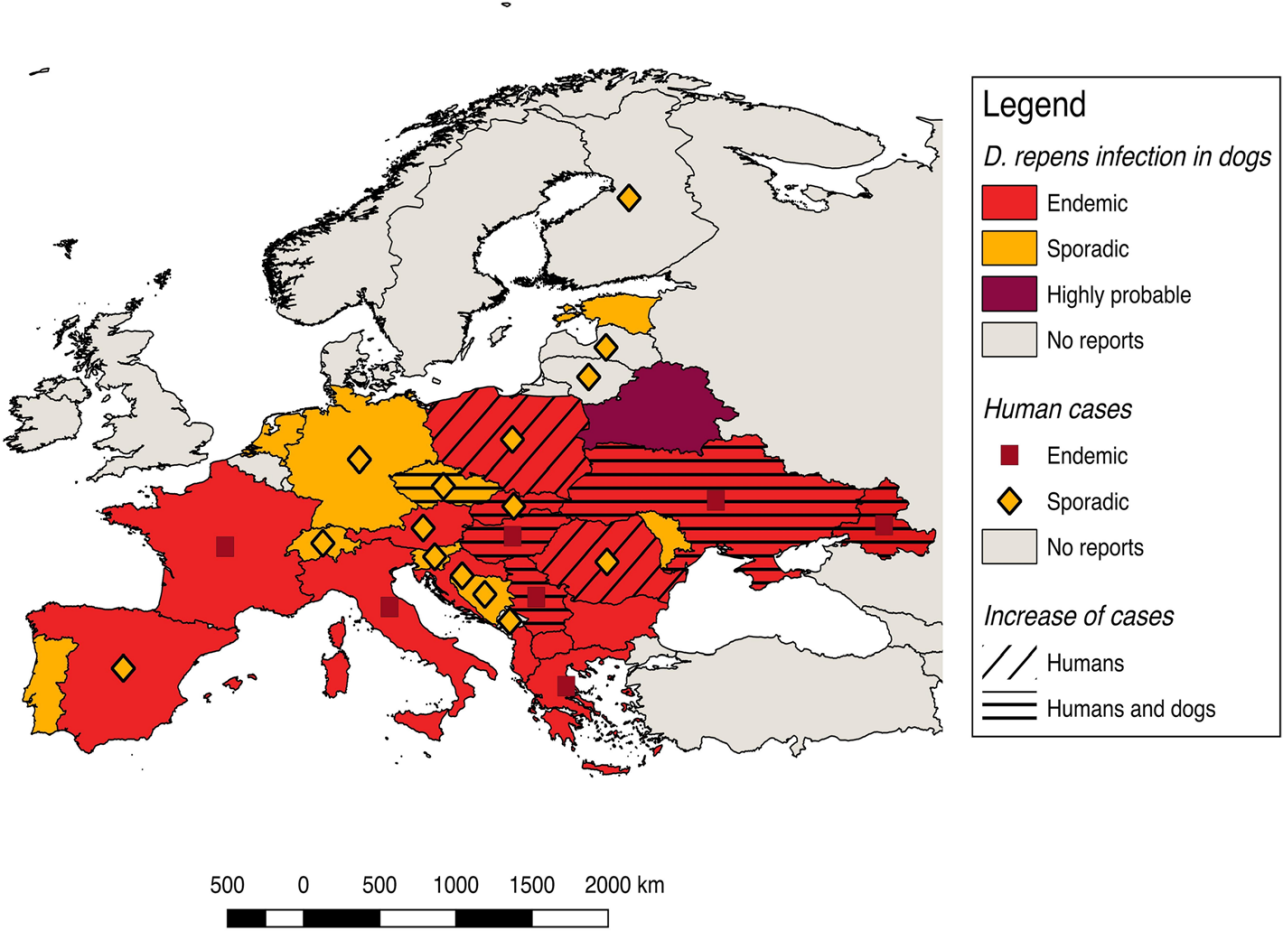
Map showing the current distribution of *Dirofilaria repens* in dogs and humans in Europe

Autochthonous *D. repens* infections have been found in dogs in most European countries, from Portugal to Russia (Fig. 1). Accordingly, human cases of dirofilariosis occur in the same areas where the infection is endemic in dogs [7] and their distribution has been previously reviewed [7, 9, 25–28]. The highest incidence of human cases has been recorded in the Mediterranean countries (Italy, southern France, Greece) and in the last two decades in some eastern European countries, namely Ukraine, Russian Federation and Belarus [7, 13, 29]. Nonetheless, many human cases are not published and the overall picture of the distribution of human dirofilariosis remains uncertain.

In the following chapters we briefly summarize and update the current distribution of *D. repens* in dogs, humans and mosquitoes in Europe, which has been divided into four zones following the Köppen-Geiger Climate Classification [30] (available at: http://koeppen-geiger.vu-wien.ac.at/pdf/kottek_et_al_2006_A4.pdf), namely Mediterranean countries (Portugal, Spain, southern France, southern Italy, and Greece), west-central and Balkan countries (northern Italy, central and northern France, UK, Belgium, Denmark, Netherlands, Germany, Switzerland, Austria, Czech Republic, Poland, Hungary, Bulgaria), eastern countries (Slovakia, Romania, Moldova, Ukraine, Belarus, Russian Federation, Lithuania, Estonia, Latvia), and Nordic countries (Norway, Sweden, Finland). Countries falling into different climatic zones have been placed in the one covering the majority of the area. Reports from other countries bordering Europe or the Mediterranean basin are also briefly mentioned.

### Mediterranean countries

In Italy, the first extensive data of canine *D. repens* prevalence were obtained in the north of the country in the second half of the last century [31, 32]. Interestingly, the results showed a higher prevalence of *D. repens* compared to *D. immitis* (30 *vs* 5% respectively) [31, 32], while 25 years later, surveys in the same areas showed a dramatic increase of *D. immitis* in dogs (20–40%) [33]. The most recent data indicate that *D. repens* is practically endemic in all of the peninsula and the major islands (Sicily and Sardinia) with a prevalence ranging between 1.5–12% [34–37], and that dogs are often co-infected with other filarioids, such as *Acanthocheilonema reconditum* and *D. immitis* [38–40]. *Dirofilaria repens* was also found in the mosquito species *Culex pipiens* in the northeastern part of the country [41], with an infection rate ranging between 0.23–0.71%.

Accordingly, Italy is one of the countries with the most significant number of human cases [1, 8, 9, 42], and case series of up to 60 patients were published [8]. A spatial correlation has been observed, with human cases reported more frequently in areas where *D. repens* infection in dogs is highly endemic [43, 44]. For example, out of 14 cases of human ocular dirofilariosis reported in Sicily (southern Italy), eight (57.1%) occurred in the Province of Trapani where the infection rate in dogs was as high as 20.4% [45].

Canine filariosis, caused by *D. repens*, has been documented in dogs from continental Spain and the Balearic Islands. In a study conducted in Salamanca Province (north-west Spain), blood samples from 293 dogs revealed *D. repens* in 0.3% of the animals [25]. A similar prevalence (0.2%) was obtained after examining 1683 dogs from three areas on the Mediterranean coastline of Spain and one in the Madrid Province (central Spain) [46]. In southeastern Spain, the presence of *D. repens* infection was evaluated in 114 kenneled dogs with the highest prevalence of infection (84.6%) observed in the Alicante Province [47].

Although Spain is frequently the country of origin for human infections diagnosed in Norway, Slovenia, Netherlands and UK [48], few autochthonous human cases have been reported, namely on the island of Ibiza [49] and in the Province of Alicante [50].

In Portugal, canine or other animal cases of *D. repens* infection have not been reported until very recently, when the first case of canine infection was found in the Algarve, the southernmost part of the country [51]. Currently there are no reports of human infection, apart from the description of an imported case [52].

Dirofilariosis is a common parasitic disease of dogs in Greece, with a higher prevalence of *D. repens* in northern Greece (30%) [53] compared to southern Greece (0.68%) [54]. The infection is also expanding in the western province (Achaia), where a positive dog was recently recorded for the first time [55]. Therefore, it is not surprising that human infections in Greece have been reported since the year 2000 [56] both in residents and tourists [57].

### West-central and Balkan countries

In France, *D. repens* has only recently received attention. Epidemiological studies conducted on military dogs in the southeast of France in 1986 and 1990 [58], showed a wider distribution of *D. repens* in comparison to *D. immitis.* A national survey on *Dirofilaria* infections seen in veterinary clinics conducted in 2006 [59] pointed out that at least one case of canine cutaneous dirofilariosis was diagnosed in 8.5% of the clinics. In general, the case frequency was considered relatively stable in the ten-year period 1996–2006, with a national average annual clinical prevalence calculated to be 0.005%. The majority of cases (74.4%) were considered autochthonous in the sampling area. The parasite was mainly distributed in the southern (Mediterranean), central and western (Atlantic) parts of the country [59].

A review of human cases reported in France during the period of 1923–1999 counted 75 descriptions, mainly from the southeastern part of the country [60]. Since then, another five cases have been described, including apparently new areas, resulting in a cumulative total of 80 cases until 2007. Interestingly, *D. repens* has been observed in 22 (23.5%) departments of France, most of them overlapping with those where canine filariosis was previously reported [58–67]. On the island of Corsica, human cases have been reported since 1994 [68] and DNA of *D. repens* has been recently found in 1.5% of *Aedes albopictus* mosquitoes [69].

The first empirical evidence of northern spreading of *Dirofilaria* infections over the Alps was in a dog from southern Switzerland at the end of the last century [70]. A few years later, another two positive dogs were found in Canton Ticino, the region bordering northern Italy [71]. Considering the close proximity of Switzerland to hotspots in Italy, it is not surprising to find some human infections in this area [72].

Other cases of possibly autochthonous *D. repens* infections in dogs of central Europe are reported from Germany [73–76]. However, the screening of 1023 blood samples collected in 2013 and 2014 in Brandenburg (north-eastern Germany) did not provide any evidence for autochthonous *D. repens* infections [77]. The finding of *D. repens* in the mosquito species *Culiseta annulata*, *Anopheles maculipennis* (*sensu lato*), *Aedes vexans* [78, 79] and *Anopheles daciae* [80], along with an analysis of weather data, suggests that active transmission within the area may occur [81]. Accordingly, in 2014 the first autochthonous human case was reported in Germany [82].

A single autochthonous case of *D. repens* infection in a dog was reported in the Netherlands in 2008 [83].

In Austria, a recent review of cases occurring from 1978 to 2014 found autochthonous *D. repens* infection in seven dogs [28]. The first autochthonous human case was described in 2008 [84]. The finding of the nematode in the mosquitoes *An. maculipennis* (*s.l.*) and *Anopheles algeriensis* [85] suggest the endemisation of the infection as well as the introduction of *D. repens* from eastern neighboring countries.

In Poland, the first foci of canine *D. repens* infection were signaled in 2009 with a high mean prevalence of 37.5% [86]. A survey conducted between 2011 and 2013 on 1588 dogs originating from all 16 provinces of Poland, revealed a nationwide distribution, with an overall prevalence of 11.7% and local values ranging from 1.2 to 25.8% [87]. A high prevalence (38%) was recently confirmed in dogs in central Poland [88]. The first human autochthonous case was published in 2008 [89], then a retrospective survey on affected human tissues since 2007 revealed a total of 18 cases of *D. repens* infections in Poland [90].

In the Czech Republic, *D. repens* occurs only in lowlands in the south-east of the country, in the triangle between the rivers Dyje (= Thaya) and Morava [91, 92], with indication of recent movement northwards along the River Morava (Modrý et al., unpublished). Recently, a report on emergence of autochthonous human infections in the Czech Republic was published, geographically overlapping with known distribution of *D. repens* in dogs [93].

In Hungary, the first dog with an autochthonous *D. repens* infection was diagnosed in 1995 [94]. An epidemiological survey carried out during 2005–2006 revealed a prevalence of 14% in dogs [95]. In the following years the national prevalence of *D. repens* microfilaremic dogs was 18%, with significant local variations of prevalence up to 30%. [96]. Accordingly, human cases are increasingly reported and *D. repens* infection is considered an emerging zoonosis in Hungary [97–101].

Cases of *D. repens* in dogs are reported in the whole Balkan region [27], with high variations of prevalence according to the area and the type of study, such as 14–47.3% in Croatia, 11% in Albania and Kosovo, 1.9% in Bosnia and Herzegovina and 21% in Macedonia (FYROM) [27, 102, 103].

Although prevalence surveys are not available for Slovenia, the parasite was diagnosed in a dog as an imported case to Germany [104].

One of the most affected countries in the Balkan area is Serbia, where *D. repens* has been found in dogs, with prevalence ranging from 17 to 49% [105]. Infection was also found to be prevalent in wild canids [106]. *Dirofilaria repens* has repeatedly been reported in humans [106–108] and a recent survey on canine and human cases revealed an endemic status of dirofilariosis in parts of Serbia [109].

Human cases are also reported in Croatia [110–112] and more rarely in Bosnia and Herzegovina [113], in Montenegro [107, 114] and in Slovenia [13]. The infection by *D. repens* in dogs of the Balkan countries is currently considered in expansion and human cases are correspondingly reported [110].

Studies performed in dogs in Bulgaria reported two positive (1%) out of 192 stray dogs [115], while in Sofia ten years later (2005–2007), 18 (4.8%) dogs out of 378 were found microfilaremic [116]. The analysis of data for a 39-year period found 47 cases of human dirofilariosis with various organ localizations [116].

### Eastern countries

In Slovakia the first microfilaremic dogs for both *Dirofilaria* species were identified in 2005 during routine blood testing [117]. The first systematic research detected microfilariae of *D. repens* in 99/287 (34.5%) dogs, confirming the country as a new endemic area of central Europe [118, 119].

In 2007 the first human case was also detected in Slovakia [120], two years after the first case in dogs. Since then, a total of 12 human cases have been registered at the Institute of Parasitology, Slovak Academy of Sciences [121–123]. The majority of cases came from the southern regions of the country, bordering Austria and Hungary [123]. Recently, *D. repens* was identified in *Anopheles messeae* and unidentified mosquitoes of the *An. maculipennis* and *Cx. pipiens* complexes [124].

In Romania *D. repens* was mentioned in dogs during expeditions that took place in1963–1964 [125]. In 2008, adult *D. repens* were found in a dog from the northeastern part of the country [126]. In the western counties, the prevalence of infection ranged between 2.2–7.2%, close to the Hungarian border [127, 128]. In a recent survey focused mainly on the southern parts of the country, the highest prevalence (18.8%) was recorded in the Danube Delta (southeast), while in the southwestern counties the prevalence values ranged between 2.2–13.4%, near the Danube [129].

The first human case report in Romania was published in 2009 [130], followed by a few other reports [131–133]. It may be assumed that *D. repens* is endemic in Romania and that a considerable number of human and canine cases remain undetected.

In the former USSR, first records of *D. repens* infection in dogs originating from Ukraine and the Rostov region of Russia were reported in the first half of the 20th century [134]. More recently (2002–2009), 20.25% of tested dogs were positive for *Dirofilaria* spp. microfilariae in the Rostov region, with *D. repens* single infection (44.7%) superseding mixed infections with *D. immitis* (25%) [135]. A large-scale survey conducted between 1995 and 2012 on 3258 canine blood samples revealed a prevalence of *D. repens* infection ranging between 10–43% in southern Russia, and up to 12% and 36% in pet dogs and service dogs of northern regions, respectively [136]. Between 2000 and 2002, a similar prevalence was recorded in Kiev (Ukraine), with 30% and 22% of stray and owned dogs, respectively, being positive. More recently, similar rates (18%) were found in client-owned dogs in Kiev [137].

In southern Russia and Ukraine, *D. repens* in humans is endemic and well known by local physicians [136, 138–148]. Of 264 cases of human dirofilariosis recorded in Russia between 1915 and 2001, 43% occurred during the last three years of the period analyzed (1999–2001) [149]. According to a genetic analysis of strains isolated from patients who acquired infection in Ukraine, there are only negligible genetic differences as compared to strains from southern Europe [150]. A recent analysis of 266 cases detected in Rostov-on-Don, Russia from 2000 to 2016 reports a relatively high proportion (10%) of mature females [151].

In various territories of Russia, infection prevalence within 6232 mosquitoes of the genera *Anopheles*, *Aedes* and *Culex* ranged between 1–14% [137]. *Dirofilaria repens* has also been found in 1% of mosquitoes collected in Tula region, in the species *Ae. vexans*, *Aedes geniculatus*, *Aedes cantans* and *Cx. pipiens* [152]*.*

In Moldova, few human cases were reported, but the finding of DNA of *D. repens* in mosquitoes from 13 of 25 trapping sites and the suitability of temperature conditions for transmission of *Dirofilaria* spp. within the entire country suggest an endemic status [153]. Indeed from 2010 to 2015, the highest infection rate of *D. repens* (4.91%) was found in *An. maculipennis* (*s.l.*), whereas the most frequent mosquito species *Cx. pipiens* (*s.l.*)*/Cx. torrentium* had significantly lower infection rates (0.88%) [153].

Thus far, the northernmost European site where the parasite life-cycle has been confirmed is Estonia (Tartu 58°23'N, 26°43'E) where *D. repens* microfilariae were reported in three dogs in 2013–2014 [154], while no human cases have been suspected or confirmed.

A human case was diagnosed after surgery in 2011 in Latvia [155].

### Scandinavia

In 2016, a survey of 125 veterinarians in the Baltic (Estonia, Latvia, and Lithuania) and the Nordic countries (Denmark, Finland, Iceland, Norway and Sweden) interviewed by a questionnaire on the presence of canine babesiosis, *D. immitis* and *D. repens*, suggested that autochthonous cases of the three vector-borne parasitic infections occur in the region [156]. Accordingly, an autochthonous human case has been diagnosed in Finland in 2015 [157].

### Other countries

Autochthonous *D. repens* infections have been reported in both dogs and humans in Egypt [158], Tunisia [159], Israel [160, 161], Iraq [162], Saudi Arabia [163], Dubai [164], Kuwait [165], Iran [166] and Turkey [167, 168]. While *D. immitis* is apparently absent from some Middle Eastern countries such as Israel where *D. repens* is present, *D. immitis* seems to be more common in dogs than *D. repens* in other countries such as Iran and Turkey [169, 170].

### Imported human cases in central and northern Europe

Most cases reported in central and northern Europe have been seen in travelers to endemic areas or in migrants. Most infections are acquired in southern Europe (e.g. Italy, Spain, Greece) and to a considerable extent in southern regions of Russia and Ukraine. However, infections are further imported from non-European countries, especially India and Sri Lanka. Interestingly, molecular analysis of human cases imported from India repeatedly revealed these as caused by *Dirofilaria* sp. “hongkongensis”, which is closely related to *D. repens* [171, 172]. Thus, cases from Asia, attributed to *D. repens* in the past, may indeed have been caused by *Dirofilaria* sp. “hongkongensis”.

Additionally, human cases of *D. repens* were repeatedly diagnosed from travelers returning from Africa, including cases from countries with no previous information on the presence of *D. repens* (e.g. Senegal and Namibia; unpublished experience of the authors).

## Life-cycle

**Fig. 2:**
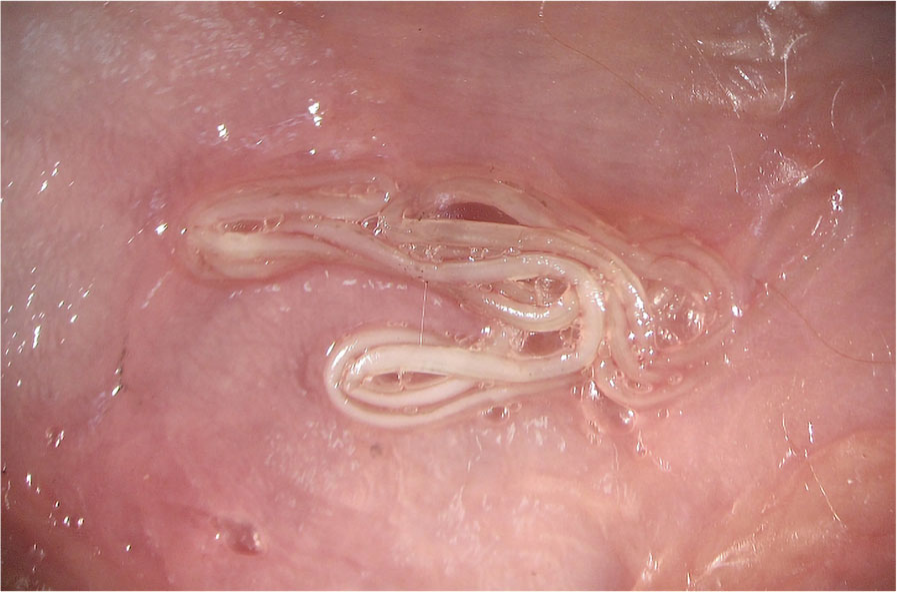
Adult specimen of *Dirofilaria repens* detected in the subcutaneous tissue of a dog during a necropsy (courtesy of Riccardo Paolo Lia)

**Fig. 3:**
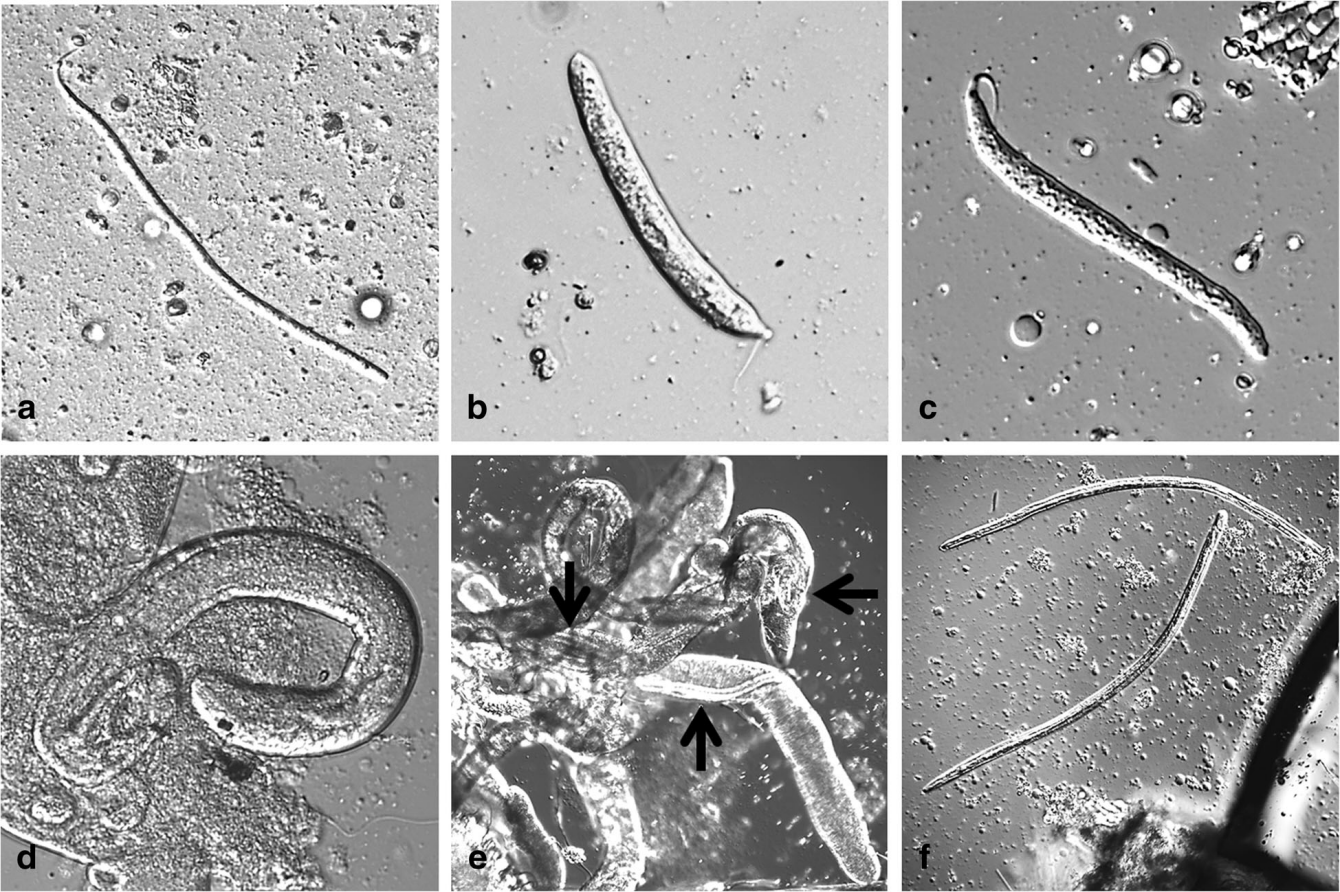
Developmental stages of *Dirofilaria repens* inside a mosquito (*Aedes vexans*) (courtesy of Cornelia Silaghi). **a** L1 day 2 pi; 335 × 9 μm, the stage still resembling a microfilaria. **b** L1 day 3 pI; 167 (214) × 18 μm, so-called sausage stage. **c** L1 day 5 pi; 198 (220) × 16.8 μm, so-called sausage stage, but more elongated. **d** L2 day 7 pi; 425 × 35 μm. **e** L2 late stage or L3 inside Malpighian tubules (black arrows), day 19 pi. **f** L3 day 16 pi, transition from thorax to head; 962 × 30 and 934 × 23 μm

*Dirofilaria repens* worms are parasites of subcutaneous and intramuscular connective tissues of dogs and other carnivores (e.g. foxes, wolves and coyotes) (Fig. 2). The females of *D. repens* are viviparous and after mating, microfilariae are released in the peripheral blood and are picked up by a mosquito, the intermediate host, during the blood meal. Soon after ingestion, microfilariae migrate from the midgut to the Malpighian tubules through the haemocoel of the insect, where they molt into the second (L2) and third (L3) infective larval stages (Fig. 3). The L3 then actively leave the Malpighian tubules to migrate through the body cavity and the thorax to the head and finally the proboscis where they wait until they are transmitted to the next host. The developmental process is temperature-dependent and takes about 8–13 days at 27–30 °C, 10–12 days at 24–26 °C and 16–20 days at 22 °C [173–175]. A delay of four days has been observed in the development at 22.5 °C and 29.4% relative humidity (RH) in comparison to 24.5 °C and 80.9% RH [174, 176]. At 18 °C, the development needs 28 days [173, 175, 177]. In the mammalian host, the L3 migrate to the subcutaneous tissue and undergo two additional molts (from L3 to L4 and to preadult worms), finally maturing into adults. In dogs, the prepatent period is 189–239 days [175], although in a recent study the first microfilariae were found in the bloodstream on day 164 post-infection (pi) [178]. *Dirofilaria repens* nematodes may live up to ten years (on average two to four years) and females potentially produce microfilariae throughout their lifespan [4].

## Epidemiology

### Vectors and transmission

In Europe, the known vectors of *D. repens* are mosquito species of the genera *Anopheles*, *Aedes*, *Culex* and *Coquillettidia*, with *Culex pipiens pipiens* [28, 41, 177, 179, 180] and *Aedes albopictus* implicated as the main vectors in southern Europe [177, 179, 181]. In central Europe, *Ae. vexans* and mosquitoes of the *Cx. pipiens* complex may readily act as potential vectors [41, 182–184].

Other mosquito species indigenous to Europe are indicated as possible vectors in nature: *An. algeriensis* [185], *An. daciae* [186], *An. maculipennis* (*s.l.*) [79, 182, 185], *Ae. caspius* [179] and *Cs. annulata* [79]. Recent studies conducted in highly endemic areas in southern Hungary and northeastern Italy have shown that the molecular screening of blood-fed or host-seeking mosquitoes is an adequate tool to verify the presence of *D. repens* and other mosquito-transmitted filarioid helminths in a certain area [41, 182]. However, the simple detection of filarial DNA is not enough to confirm the occurrence of microfilariae development into infective L3 stages. Filarial DNA must be detected in separate body regions of the mosquito and the positivity of the head/thorax samples can indicate that infective larval stages had developed within the mosquito host [177, 180, 181].

### Vector competence

Several factors define the vectorial capacity of a mosquito species for a specific pathogen: vector competence (i.e. the percentage of vector individuals able to support the development to the infective stage), mosquito density and seasonality, extrinsic incubation time, host preference and daily biting rate, expected infective lifetime, the mosquito daily survival rate, as well as the availability and density of infected vertebrate hosts [80, 81, 187]. For the successful transmission of *D. repens* L3 to a canine (or other vertebrate) host, an infected mosquito must survive for at least the extrinsic incubation time until the highly motile L3 have reached the proboscis. Furthermore, the mosquito species needs to be endemic at localities where dogs are present to acquire and transmit the infection, and it needs to have a particular biting preference for canines. Therefore, this renders mosquito species with a mammalian host preference present in urban and suburban localities suitable for the support of an endemic *D. repens* cycle.

The vector competence of several mosquito species for *D. repens* has been shown in experimental laboratory studies by observation of the development to the infective L3 stage: *Ae. aegypti* [15, 174, 176, 188]; *Ae. albopictus* [189]; *Ae. caspius*, *Aedes detritus* [173]; *Aedes mariae* [174]; *Ae. vexans*, *Anopheles stephensi* [175]; *Anopheles claviger*; *An. atroparvus* [175]; *Anopheles sinensis* [174]; *Culex pipiens molestus* [188]; *Aedes togoi* [190]; *Ae. geniculatus*; and *Aedes japonicus* [191]. Different methods for the infection of the mosquitoes were applied in these studies such as the direct feeding on a microfilaraemic animal [173, 176, 188] or the artificial membrane feeding with infected blood [192].

Furthermore, within a certain species of mosquitoes, susceptibility or refractoriness may vary considerably and may be dependent on certain genes, as has been shown for *Ae. aegypti* [193]. Controversial results exist also for *Cx. pipiens*, as it has been shown both susceptible and refractory in laboratory experiments [176]. This might be attributed to testing of different biotypes (pipiens, molestus and their hybrids) that possess different vectorial capacity. *Culex pipiens fatigans*, *Anopheles gambiae* complex, *Aedes vittatus*, *Ae. aegypti* and *Mansonia africana* were also shown to be refractory to *D. repens* infection in laboratory investigations [176, 191]. All microfilariae in the latter mosquito species were trapped inside the midgut in the blood clot and were disintegrated and no longer observable after day 5 pi. This retention of microfilariae has been described as potentially beneficial to the vector-parasite interaction system. A reduced microfilarial burden can lead to increased mosquito longevity, potentially making it more efficient transmitting host [194]. Microfilaria burden can vary greatly in a canine host and consequently also the uptake of microfilariae by a mosquito vector. This variation may be due to the circadian rhythms of microfilariae in the peripheral blood and mosquito vector biting [6, 175].

Apart from the process of microfilaria degradation and melanisation as part of an innate immune response of the mosquito host [195], it was also assumed that the anatomical structures of the alimentary channel and the physiology of the respective mosquito species influence the development of microfilariae, for example the speed of blood clotting after blood intake (discussed in [188]). Some authors have highlighted the importance of mosquito cibarial armature and peritrophic membrane in the transmission of *D. repens*. Indeed, cibarial armature and dome can mechanically damage a high proportion of microfilariae, which are ingested with the blood meal, and possibly serve to protect mosquitoes [188, 189]. Development and complexity of the cibarial armature differ between different species. In some it is absent (*An. atroparvus*, *An. claviger*, *Ae. aegypti* and *Ae. mariae*), in others it has one (*Anopheles albimanus* and *Anopheles farauti*) or two (*An. gambiae*, *Anopheles stephensi* and *Anopheles superpictus*) rows of cibarial teeth, whereas in *Cx. p. pipiens* teeth of cibarial armature are spoon-shaped and the cibarial dome is strongly denticulated [196, 197]. The number of damaged erythrocytes varied between 2–4% in the first, and 45–50% in the last group. The time needed for formation of peritrophic membranes in adult mosquito varies between 4 and 12 h in different species [198].

### Risk factors

No study has been published on risk factor analyses using a multivariate approach, which would be more suitable for highlighting confounding factors and biases. Therefore, some of the associations found and often reported as risk factors (Table 1) are likely the results of the interaction of different factors related to the host (sex, age, breed and lifestyle), the vector (presence, density, vectorial capacity and attraction to dogs), the environment (rural, urban, climate) and the human intervention (use of specific chemoprophylaxis and/or physical or chemical protection against mosquitoes).

**Table 1:** Factors significantly associated with *Dirofilaria repens* prevalence in dogs of Europe

No. of dogs tested	Country	Potential risk factors	Reference
114	Southern Spain	Kenneled dogs (lack of preventative measures)	[47]
		Geographical location	
2406	Central Italy	Older age	[294]
		Male sex	
		Pure breed	
		Traveling dogs	
151	Eastern Slovakia	Older age	[119]
		Lifestyle (outdoors)	
		Geographical location	
972	Central Italy	Rural environment	[34]
		Geographical location	
194	Northern Serbia	Older age	[295]
		Geographical location	
2512	Southern Italy	Lifestyle (guard dogs)	[296]
		Geographical location	

The evaluation of the frequency of the factors associated with *D. repens* prevalence in literature, in particular male and guard dogs, older age and outdoor lifestyle, suggests that the higher exposure to mosquito bites is the only risk factor clearly associated with *D. repens* prevalence.

## Canine subcutaneous dirofilariosis

Although canine *D. repens* infections very often runs asymptomatically, a plethora of nonspecific dermal alterations has been reported such as skin nodules, pruritus, thinning, itching and asthenia [10, 59, 199, 200]. Usually, no inflammatory reaction or connective capsules are surrounding the living parasite (Fig. 2a), which can be seen moving actively under the connective serous layers [4]. Non-inflammatory subcutaneous nodules, cold, not painful and mobile, can be seen on the skin surface of infected animals. Inflammatory and painful nodules may be associated with localizations such as the scrotum. Granulomatous capsules generally surround dying and degenerating worms. These clinical alterations, however, must be supported by histopathological data or *D. repens* microfilaria-positive blood examinations or molecular identification from biopsy. Lesions may also appear as circular alopecic areas with lichenification, hyperpigmentation and erythematous and scaling margins [201] and they can occur in the lumbosacral and perianal regions [164]. Skin affections may be pruritic or not, suggesting that itching is not crucial for a presumptive diagnosis of *D. repens*-associated dermatitis. An unusual case of allergic non-pruritic diffused dermatitis caused by *D. repens*, confirmed by histological examination, has also been described [201].

*Dirofilaria repens* infection was the aetiological cause of ocular lesions in a dog reporting conjunctivitis and later additional ocular and nasal mucopurulent discharge [202]. Worms were then found in a dorsonasal bulbar conjunctival mass and in the ventral palpebral conjunctival fornix and confirmed as *D. repens* by PCR. Rarely, *D. repens* may reach ectopic body parts. A case of adults in the pelvic cavity and mesentery was reported in a dog with a diagnosis of kidney failure and chronical cystitis [203].

The histological examination of lesions may reveal the presence of multifocal purulent dermatitis, panniculitis, hyper-pigmentation and hyperkeratosis [10]. Generalized cardio-hepato-renal insufficiency may also occur [87]. Pathological changes are most likely associated with the presence of adult nematodes or microfilariae [10]; however, symbiotic *Wolbachia* bacteria, which live in the hypodermal chords of *Dirofilaria* male and female adults, and in the female germline [204], have been shown to increase the level of pro-inflammatory cytokine (e.g. IL-8) and induce chemoattraction [205, 206].

## Human infections

Humans acquire the infection in the same manner as dogs, by the bite of a mosquito, but it is probable that most of the infective larvae die shortly after, with the infection resolving unrecognized and without causing any specific symptom [1, 8]. No predisposing factors are known to explain why in some cases larvae may develop further. After the bite of an infective mosquito, a stronger reaction with erythema, swelling and pruritus lasting 5–8 days is reported [1, 8]. In most of the cases a single worm develops, probably because the stimulation of the immune system prevents the development of others [1, 8]. In rare cases the worm may develop to a mature adult [1, 207, 208] and even fertilized worms releasing microfilariae have been described, especially in immunosuppressed patients [1, 8, 42, 146, 209–212], which in very rare cases may even reach the bloodstream [213].

**Fig. 4:**
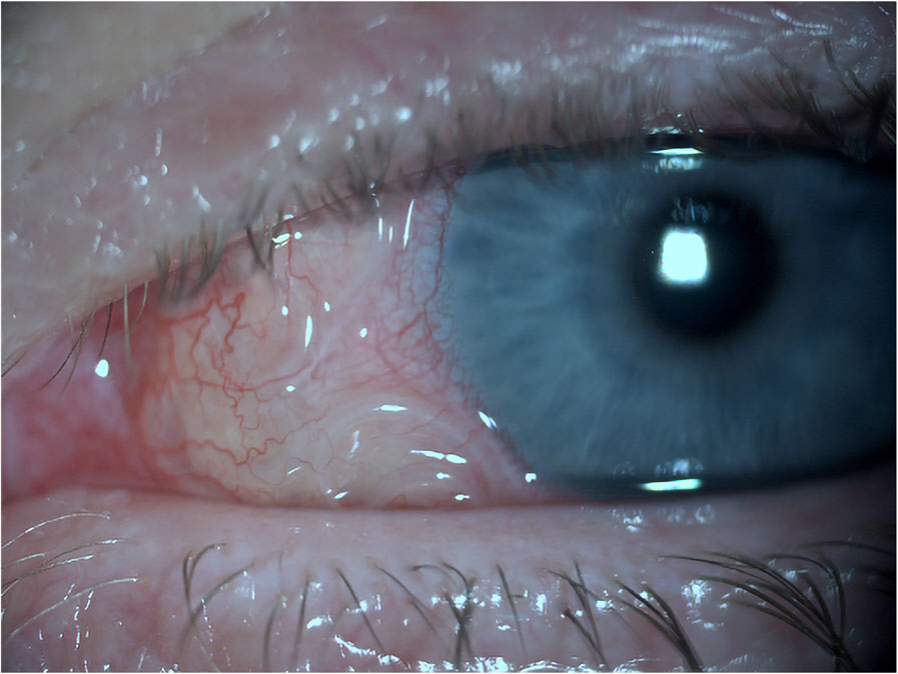
*Dirofilaria repens* visible in the subconjunctiva of a human eye (courtesy of Ramin Khoramnia and Aharon Wegner)

In infected patients, the developing stages of *D. repens* migrate subcutaneously [1, 8, 61] for weeks up to several months in several parts of the body, usually with mild and unrecognized symptoms [1, 8, 61] and only sometimes causing larva migrans-like symptoms (i.e. irritation and itching) [1, 8, 42, 61, 131, 211, 214]. In one case, a patient, after scratching a pruritic lesion, removed a 6 cm long whitish worm from the wound [215]. During migration *D. repens* may reach the eyes [1, 8, 61, 211], becoming visible through the subconjunctiva [1, 5, 72, 110, 113, 168, 214, 216–219] (Fig. 4). Larval stages localized in the eyes can be removed surgically without serious damage [1, 214, 219]. However, in rare cases, serious sequelae (glaucoma, uveitis, episcleritis and retina-detachment) may develop and ultimately lead to significant loss of vision [1, 8, 100, 147, 220–222].

After weeks to several months from the infection, *D. repens* may stop to migrate and form a nodule of about one centimeter [1, 8]. In most cases, the nodules develop subcutaneously [1, 8, 48, 63, 93, 108, 111, 116, 138, 158, 212, 223–228]. Nodules have been reported in various human body areas and tissues, mostly in the superficial tissues of the facial regions [1, 8], as perioral and periorbital tissues [107, 167, 224, 226, 227, 229–234], forehead [235], skin of the lower leg [93], soft tissues of the hand [236] or finger [93], subcutaneous tissue of the hypogastrium [93] and of the neck [237]. Other predilection sites are scrotum and testicles and, to a lesser extent, the breasts of women [1, 8, 65, 223, 235, 238–245]. Various reasons have been hypothesized for these preferences, such as lower body temperature of these areas, higher awareness of patients for these body parts or a tropism of *D. repens* to higher concentrations of sexual hormones [1].

The nematodes may also reach deeper body areas, such as lymph nodes [93], the abdominal cavity [93, 99], lungs [1, 56, 158, 246], muscles [247] and even the dura [64].

If left untreated, *D. repens* may survive for up to one and a half years [1, 8]. The symptoms caused by *D. repens* nodules depend on their localization, usually being limited to a local irritation, erythema and pruritus [1, 8, 93]. Rarely, a strong local immune reaction develops, and the nodules may appear like a suppurating abscess with local infection accompanied by a mild systemic reaction, including elevation of body temperature and mild eosinophilia [1, 8, 206]. In very rare cases, even more severe systemic immunoreactions may develop, manifesting as fever or lymphadenopathy. A case of meningoencephalitis has also been reported [211]. Comparatively severe symptoms are seen in immunosuppressed patients and in the rare cases where microfilariae develop [1, 8].

## Diagnosis in dogs

**Fig. 5:**
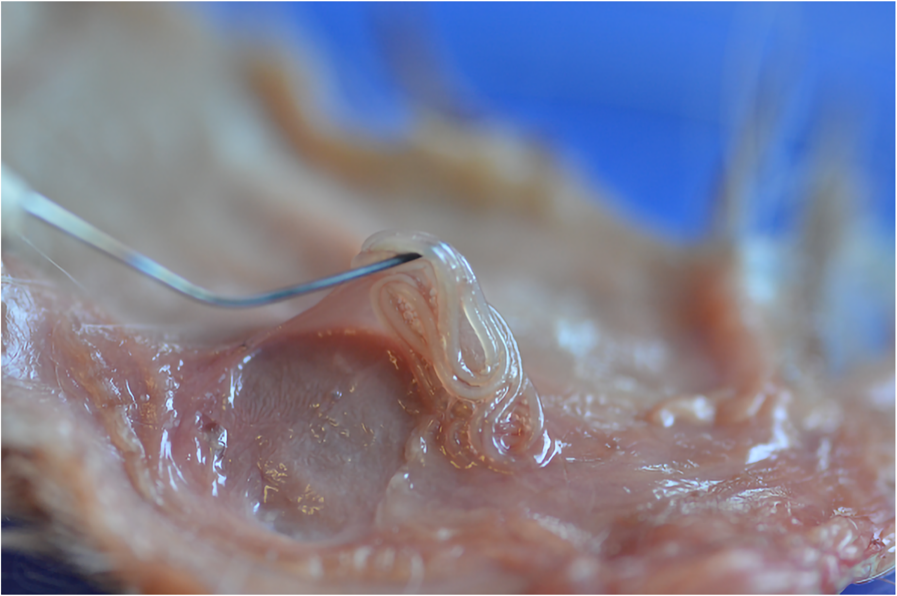
Adult *Dirofilaria repens* removed from the subcutaneous tissue of a dog during necropsy (courtesy of Riccardo Paolo Lia)

Diagnosis of *D. repens* may be performed by detection and identification of circulating microfilariae, morphological and molecular identification of adult parasites, cytological examination of fine-needle aspiration biopsies and histopathological examination of excised nodules. In the case of localized skin lesions, the adult nematodes can be recovered from the nodules located in different anatomical sites of the animal (e.g. chest or lower limbs) [10] (Fig. 5), while in cases of localized or generalized dermatitis adults are almost impossible to find.

**Fig. 6:**
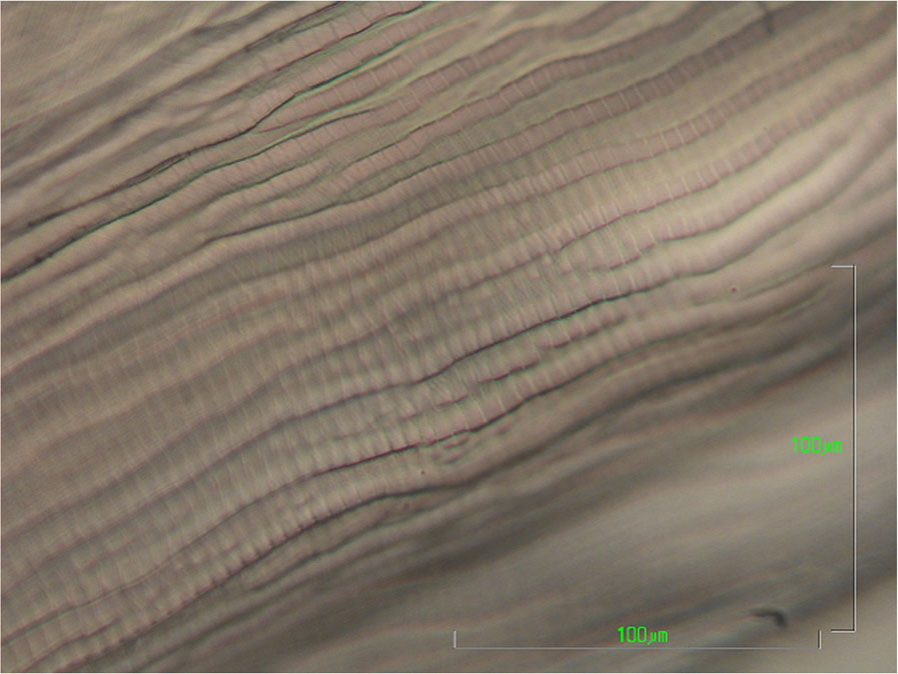
Aspect of the ridges of the cuticle of *Dirofilaria repens* under scanning electron microscopy (courtesy of Sven Poppert). *Scale-bars*: 100 μm

**Fig. 7:**
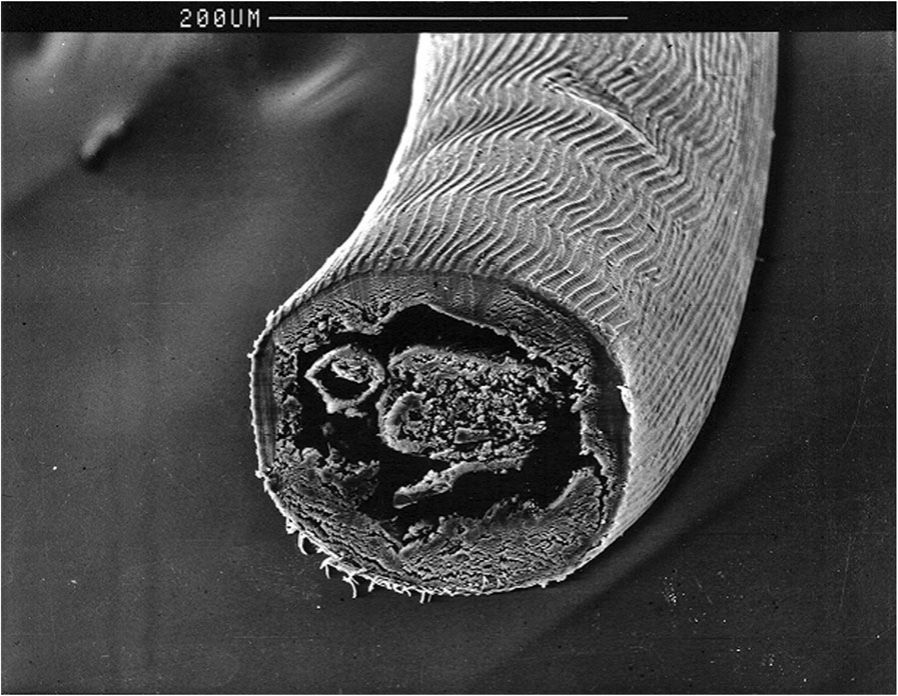
Cuticle morphology of *Dirofilaria repens* under scanning electron microscopy (courtesy of Salvatore Giannetto). *Scale-bar*: 200 μm

On gross examination, the cuticle of *D. repens* specimens is whitish, with distinct longitudinal ridges on the surface (Figs. 6 and 7), and narrows at the ends. Males measure 48–70 mm in length and 3.7–4.5 mm in width, while the females are larger, reaching 100–170 mm in length and of 4.6–6.5 mm in width [248, 249]. Upon accurate microscopic observations, the clarification of specimens with lactophenol or with glycerine for temporary mounts, allows the observation of distinct morphological features, such as the vagina in the female, which opens at 1.1–1.9 mm from the oral aperture, or the two spicules in the male, measuring 430–590 and 175–210 μm, respectively, as well as 4–6 precloacal papillae (1–2 post-anal and 3 caudal). In the case of adults embedded in the nodule, *D. repens* specimens are identified at the histology on the basis on their body diameter (220–600 μm), and by the presence of the longitudinal ridges, each separated from the others by a distance that is larger than the width of the actual ridge itself [250]. In transverse sections stained with haematoxylin-eosin, the occurrence of longitudinal muscles and the multilayered cuticle, expanding in the region of the two large lateral chords, is indicative for *D. repens* [10, 250]*.*

The subcutaneous nodules can be also examined by ultrasound and the parasite is visualized as double linear parallel hyperechoic structures [251].

**Fig. 8:**
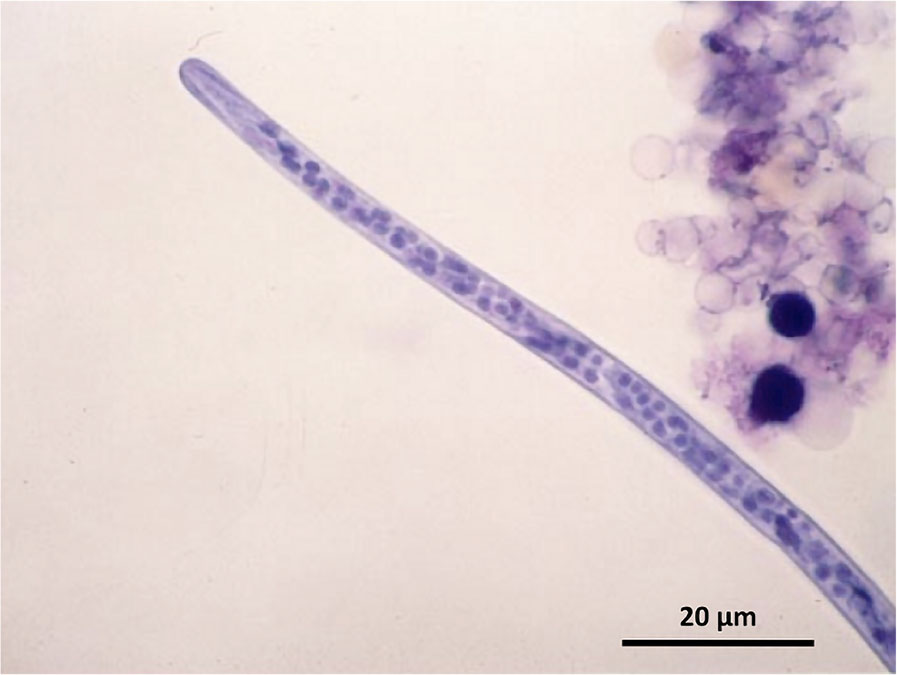
The round head of the microfilaria of *Dirofilaria repens* (Knott’s test). *Scale-bar*: 20 μm


More often the diagnosis of subcutaneous dirofilariosis is based on the visualization (see Additional file 1) and morphological identification of the blood-circulating microfilariae, by concentration methods (e.g. modified Knott’s test or filtration) (Fig. 8), histochemical staining (e.g. acid phosphatase activity) and fine needle sampling of nodules containing fertile adults. A blood sample taken in the evening may maximize the chance to find circulating microfilariae, due to the circadian variation of microfilariae in naturally infected dogs [6, 252].

**Additional file 1:** Live microfilaria of *Dirofilaria repens* in the bloodstream of a dog. This movie shows the morphology and movement of the microfilaria of *D. repens* in a direct blood smear. (MOV 9179 kb)


*Dirofilaria repens* microfilariae are unsheathed, having an obtuse-rounded cephalic margin (Fig. 5), and a long sharp tail, often curved [253, 254]. Their size may vary as a consequence of collection and fixation methods. The mean length is 300–370 μm and the mean width is 6–8 μm [253]. In a recent study [254], a mean length of 369.44 ± 10.76 μm and a mean width of 8.87 ± 0.58 μm was reported using the Knott’s test on 171 microfilaraemic dog blood samples originating from eight European countries. The test was able to clearly distinguish between *D. immitis*, *D. repens* and *Acanthocheilonema* spp. [254].

On the contrary to *D. immitis* infection, for which several, easy and rapid in-clinic test kits, based on detections of circulating antigens mainly produced by females, are commercially available for the serological diagnosis of the infection, no similar specific serological tests are available for *D. repens*.

The identification of *D. repens* may be carried out by molecular methods testing parts of adult specimens, microfilariae (in whole blood or on filter paper), or larval stages in the mosquito vectors. Various techniques have been developed for the specific detection of *D. repens*, such as multiplex PCRs targeting several filarioid species, but also for the entire superfamily Filarioidea. Amongst these are conventional and real-time PCRs, probe-based or high-resolution melting analysis techniques. The most common gene targets used are the cytochrome *c* oxidase subunit 1 (*cox*1) as a barcoding gene, the inter-genic spacer (ITS) regions, and *12S* rRNA gene [41, 184, 185, 255–259]. Other target genes used to identify the nematode are listed in Table 2. The high sensitivity of real-time PCR allows the detection of small amounts of genomic DNA either in dog blood or mosquitoes (2.5 and 0.3 pg/μl for *D. immitis* and *D. repens*, respectively) being potentially useful for epidemiological studies [41]. In addition, a multiplex PCR targeting a barcoding region within the *cox*1 gene was developed for the simultaneous detection of almost all the filarioids infecting dogs in Europe (i.e. D. *immitis*, *D. repens*, *A. reconditum* and *Cercopithifilaria* sp.) [260], therefore representing a new tool for the molecular detection and differentiation of canine filarioids in blood and skin samples. Nonetheless, positive PCR alone should not be considered sufficient to establish *D. repens* as a cause of subcutaneous nodular lesions in the absence of clear cytological picture [261].

**Table 2:** Target genes used to identify *Dirofilaria repens* in animals, humans and mosquitoes, available on GenBank (accessed 10th September 2018)

Gene	Hosts	Length (bp)
*12S* rDNA	Human, dog, cat, mosquito	116–510
*cox*1	Human, dog, cat, mosquito, beech marten	123–715
*16S* rDNA	Human, mosquito	366–487
*18S*-*5.8S*-*28S* rDNA	Human, dog, mosquito	153–2230
*18S*-small subunit ribosomal RNA	Human, dog, jackal	613–839
*hsp*70	Dog	553
*rbpI*	Dog	594
*MyoHC*	Dog	734

## Diagnosis in humans

The diagnosis of a *D. repens* infection in humans is affected by the localization of the worm and the clinical symptoms. If the infection occurs as larva migrans, especially in the subconjunctiva, and the patient was not exposed to other potential causes of larva migrans, the clinical picture is highly suggestive of *D. repens*. The anamnesis should exclude the visit of the patient to endemic areas of other filarioids, such as *Loa loa* in Africa. In case of intraocular cysts or subcutaneous nodules, the diagnosis is more difficult, but a live moving worm can be seen using a pre-operative high-resolution ultrasound [231, 245].

In most cases, the definitive diagnosis is obtained after the worm removal, using the same methods applied for animals. Microscopically, *D. repens* females do not usually contain microfilariae. The most discriminative features of *D. repens* are the longitudinal ridges of the cuticle (Figs. 6 and 7), not present in any other filarial worm infecting humans except for *Dirofilaria* sp. “hongkongensis”, a recently proposed new species from Hong Kong [262] and *Dirofilaria ursi* present in North America, North Europe and Japan in bears and rarely also in humans [171].

Since none of the described features are entirely specific, molecular tools should be applied in order to confirm the morphological diagnosis and avoid misdiagnosis, which may occur in some case with *D. immitis* [263]. In this respect, it should be suggested to surgeons to conserve the removed worm, one part in formalin for histology and another refrigerated or frozen for molecular identification. Most typical features are recognizable in histological slides, if a proper section is available and the worm not degraded. In these cases, it is still possible to perform molecular investigations from paraffin sections. An extensive description of *D. repens* in human tissue is already available [264].

Serological investigations are not helpful in human cases. In filarial infections, the immunological reaction is mainly triggered by microfilariae, which rarely develop in humans. Therefore, in most human *D. repens* cases, no antibodies against filariae are detectable or very low titers can be found [47]. However, such low titers are also seen in other nematode infections due to cross-reactive antibodies. The investigation of blood samples by microscopy or PCR is not useful for the same reason.

## Mitochondrial genotypes and potential cryptic species

A new species of *Dirofilaria* infecting dogs and humans has been first described from Hong Kong and designated as *Dirofilaria* sp. “hongkongensis” [262, 265]. This new species was proposed on the basis of relative short DNA sequences from the mitochondrial cytochrome *c* oxidase 1 and the nuclear ITS1 locus. Unfortunately, at that time all ITS1 sequences on GenBank were from *D. repens* samples collected from Thailand while all ITS2 sequences were of European origin which hampered comparisons with European *D. repens* data. Complete sequencing of mitochondrial genomes from four worms initially identified as *D. repens* using morphological features and short DNA sequences, revealed that three sequences from European samples were very similar while a fourth one collected from a patient after traveling in India was very similar to *Dirofilaria* sp. “hongkongensis” [171]. An additional *D. repens* mitochondrial genome sequence available from GenBank (accession no. KR071802) is also highly similar to the other European samples but its geographical origin is not available from the database entry. The organization of these mitochondrial genomes is identical to those of other onchocercids and like all clade III nematode mitochondrial genomes lacks the *atp-8* gene that is present in most animal mitochondrial genomes. It is slightly smaller than any of the other mitochondrial genomes described for the Onchocercidae and has the most extreme AT skew with a very high T content on the coding strand.

Phylogenetic analysis using all coding regions from the whole genomes revealed that *D. repens* and *Dirofilaria* sp. “hongkongensis” are more closely related to each other than to *D. immitis* [171]. However, as long as no other mitochondrial genomes from species of the subgenus *Nochtiella* are available, it remains speculation how closely related both species actually are. The overall similarity of mitochondrial genomes was lower than for the comparison between the human parasite *Onchocerca volvulus* and its sibling species *Onchocerca ochengi* infecting cattle. This suggests that both might represent valid species [171]. Sequencing of partial genomic fragments of approximately 2.55 kb, including the most variable long non-coding region of the mitochondrial genome, from 41 canine samples (29 from Europe and two from Thailand) and one human sample from Vietnam, revealed further heterogeneity. In the phylogram, all European and the Vietnamese sequences were located in the same statistically highly supported cluster with the complete *D. repens* mitochondrial genome sequences. With the exception of only two samples (one from Hungary and one from Poland), differences between the remaining *D. repens* sequences were small although there were some subclusters containing preferentially samples from Poland and Hungary or from southwestern Europe and Hungary in addition to a German sample. The two samples from Thailand had very similar sequences and were more similar to *Dirofilaria* sp. “hongkongensis” than to the *D. repens* cluster. However, the genetic distance between samples from India and Thailand was considerable and the latter might represent a third species [171]. These data support the view that what is currently considered to be *D. repens* is in fact a species complex with different genotypes. However, data are not sufficient yet to decide whether different genotypes from various geographical origins represent valid species, subspecies with limited geographical range or only variants within a population. Multi-locus phylogenetic analyses using samples from diverse endemic regions combined with experimental crosses would be required to define valid genospecies within the *D. repens* complex.

## Treatment and prevention

### Dogs

Due to the lack of specific clinical alterations, the treatment of *D. repens* infection in dogs often goes along with its prevention, which should be routinely performed in order to reduce the risk for the transmission to humans (Table 3). Most therapeutic protocols currently available have been translated from the experience developed in the prevention of heartworm disease and are based on the administration of macrocyclic lactones. However, contrary to heartworm disease, very few experimental studies have been carried out to assess the efficacy of macrocyclic lactones against *D. repens* [4].

**Table 3:** Macrocyclic lactones tested for the prevention of *Dirofilaria repens* infections in dogs

Active ingredient	Formulation	Dosage
Ivermectin	Tablet/Chewable	6 μg/kg
Ivermectin + Praziquantel	Chewable	6 μg/kg + 5mg/kg
Ivermectn + Doxycycline	Chewable + Tablet	6 μg/kg + 10mg/kg
Doramectin	Injectable	0.4 mg/kg
Milbemycin oxime + Praziquantel	Chewable	0.5–5 mg/kg
Moxidectin	Injectable	0.17 mg/kg
Moxidectin + Imidacloprid	Spot-on	2.5–10 mg/kg
Selamectin	Spot-on	6 mg/kg

A complete clearance of *D. repens* microfilariae was achieved in a dog treated with an off-label protocol based on melarsomine injection followed by doramectin [160], but this fact needs further confirmation since no efficacy was found in previous clinical studies [4]. Different dosages of moxidectin in oral, injectable sustained-release and spot-on formulations showed long-term suppression of *D. repens* microfilaraemia, being highly efficacious for the treatment of dogs positive for subcutaneous dirofilariosis in both natural conditions and experimental studies [266–271]. Currently, the only protocol claiming adulticidal activity for this filarioid is represented by the use of a spot-on product containing imidacloprid/moxidectin for six consecutive months, a protocol which has also been used to prevent the onset of skin lesions and dermatitis caused by the parasite [178]. Interestingly, the microfilaricidal efficacy of monthly administration of ivermectin [272] may be improved by including doxycycline [273]. This therapeutic schedule represents a novel approach for the treatment of dirofilariosis, targeting the *Wolbachia* endosymbionts of the nematode [274] and allows the reduction of the recommended ivermectin dosage, along with a minor risk of drug resistance.

As in the case of the treatment, the prevention of *D. repens* infection is largely based on the regular use of macrocyclic lactones (Table 3). When designing a rational approach for the control of dirofilariosis, the regional distribution patterns and the transmission period of the parasite should be taken into account, which derives from detailed epidemiological maps of the disease.

The prevention of *D. repens* transmission becomes increasingly important, considering that reducing the burden of canine dirofilariosis represents the only effective measure to decrease the risk for human infection, as dogs are the most important reservoir of the parasite.

Monthly applications of selamectin in a spot-on formulation were successfully used to reduce the pathogen transmission under natural field conditions for six months [275]. In addition, when infected animals were treated twice a month, the period of dog protection increased to nine months [276]. The use of moxidectin in a sustained-release formulation administered subcutaneously was found to have a complete efficacy in the prevention of *D. repens* in an experimental trial [269] and the authors suggested that the excellent action of the formulation was most likely attributed to the high lipophily of this active ingredient, which is stored in the body fat. Furthermore, moxidectin may be of great value towards the prevention of this filarial parasite and against adult parasites, when given as a spot-on treatment in combination with imidacloprid (imidacloprid 10% and moxidectin 2.5%) [40, 178].

Finally, milbemycin oxime, another macrocyclic lactone, given orally once per month also proved to be effective in protecting dogs from subcutaneous dirofilariosis in endemic areas and may offer further chemoprevention option [277].

Another important part of the prevention of infection is based on contact repellent insecticides. This can be obtained by the use of veterinary products that contain pyrethroids with a specific label on the prevention of *Culex* and/or *Aedes* bites. This prevention is particularly important in periods of activity of mosquitoes and in areas where the risk of transmission is high. The use of topic repellent may also decrease the transmission of *Dirofilaria* from infected dogs to mosquitoes [278].

### Humans

Theoretically, no special treatment is necessary in humans, because *D. repens* does not cause severe symptoms and usually dies after some time [1, 8]. The nematode can be removed by surgery, a practice that is also needed for the etiological diagnosis and to exclude other severe diseases, such as a carcinoma [1, 8]. As soon as *D. repens* has formed a stationary nodule, surgical removal can be conducted following standard procedures corresponding to the site of infection.

If a migrating *D. repens* is discovered in the conjunctiva, removal is comparably easy because the worm is visible through the conjunctiva [1, 8, 172, 214]. On the contrary, surgical removal of subcutaneous worms may be unsuccessful, due to the difficulties in precisely locating the parasite.

Medical treatment with anthelminthic drugs, such as albendazole, coupled with doxycycline, was found to stop migration of the worm and promote the formation of a fixed nodule, which can then be removed [136]. The efficacy of such treatment suggests that doxycycline may have a role targeting the endosymbiont *Wolbachia*, as has been found in dogs [274]. In addition, the immune response of humans to *Wolbachia* may be used for further confirmation of exposure to the parasite [279].

As soon as *D. repens* is removed, no further medical treatment is required, unless the patient is immunosuppressed or in the extremely rare case of a suspected second nematode [1, 8]. Because of the rareness of the disease in humans, there are no guidelines or treatment studies and the physician have to rely on their experience. However, either with or without treatment, there is not a single report of a fatality or of permanent body damage.

Prevention of dirofilariosis in humans can be achieved by protecting people from the bites of mosquitoes through the use of repellents and by reducing the prevalence of *D. repens* in dogs, the principal reservoir of the parasite [280].

## Potential drivers for the emergence of *Dirofilaria repens*

The enhanced dissemination of *D. repens* in Europe has been primarily attributed to global warming and the rapid geographical expansion of some invasive mosquitoes (and/or increases in their density), but also to increased travel and movement of infected animals into non-endemic areas along with a change in human activities [4, 11].

The effects of climate change in Europe have been extensively debated [281], since warmer climates could favor mosquito breeding and shorten extrinsic incubation periods [282], thus enhancing the risk of *Dirofilaria* spp. transmission. The projected increment in temperature will impact on insect vectors through broadening areas of colonization, invasion of new sites and, eventually, resulting in physiological changes and increased vectorial capacity. The most recent example is the finding of *Uranotaenia unguiculata*, a thermophilic mosquito species frequently occurring in the Mediterranean, in northern Germany, some 300 km north of previous collection sites [283].

An increase in mean temperatures has affected the mosquito abundance and their seasonal survival in many areas of Europe greatly impacting on the spread of filarial infestation and making most of the European countries suitable for *Dirofilaria* spp. transmission [284, 285].

A recent climatic model studied the impact of a regional warming (Russia, Ukraine, and other countries of the former USSR) on the spreading of *D. repens* and the risk of transmission to humans [26]. The model predicted an increase of 18.5% in transmission area and 10.8% in population exposure by 2030.

In addition, several intrinsic factors linked to the specific vector mosquito species also impact on the distribution of *D. repens*. The expansion of dirofilariosis somehow matched the second introduction of *Ae. albopictus* in Europe (Italy) [286]. Furthermore, over the past decades, *Cx. p. pipiens* has changed its endophagic and anthropophagic behavior in central Europe [287], where it also searches for human blood outdoors, close to the houses, as happens in southern parts of the continent.

The introduction of the Pet Travel Scheme in 2000, allowing an easier movement of companion animals throughout the European Union [288], has likely contributed to the diffusion of *D. repens* in Europe. The first case of *D. repens* in a dog resident in UK was recently reported in a dog originated from Romania and was not easily identified [202], thus reactivating the discussion on the implications for establishment and spread of *D. repens* in non-endemic countries.

Once *D. repens* has been introduced in a new area with an infected dog, the availability of suitable hosts for *D. repens*, the presence and density of competent mosquito vectors and their feeding behavior are among the most important factors impacting on its further distribution. Dogs are optimal reservoirs of *D. repens* also because they attract competent mosquito vectors and are quite tolerant to mosquito bites [11]. Prevalence of microfilaraemic dogs and presence and abundance of competent vectors also affect the rate of infestation within a given mosquito population, which in turn is directly related to the risk for a native dog to be infested.

The factors enhancing the exposure of the host to the vector (i.e. the dog’s size, the age and especially the outside exposure) may further increase the risk of *D. repens* infestation [2]. The role of cats and foxes as reservoirs is marginal, because these hosts are rarely microfilaraemic [289].

However, the general factors discussed above should have affected the emergence of both *D. repens* and *D. immitis*. Although a few reports have been published until now on the spread of *D. immitis* towards northern Europe [118, 290–292], there is no doubt that *D. repens* has spread faster than *D. immitis* from the endemic areas of southern European countries and currently it is more prevalent in northern Europe, as confirmed by the emergence of human infections (reviewed in [4, 7, 9, 27, 136]. The reasons for this could be linked to the fact that while heartworm infection causes a severe clinical condition in dogs, *D. repens* in most cases is difficult to diagnose and the course of the infection can be completely asymptomatic. As a consequence, many canine infections can run unnoticed and the infected dog continues to act as a reservoir for competent mosquitoes locally and if transported to non-endemic areas.

In addition, for heartworm infections, several rapid, easy, in clinic whole blood/serological kits are available that detect the circulating antigens of female worms. This allows veterinarians a prompt diagnosis while no serological diagnostic is commercially available for *D. repens*, hampering a rapid screening in dog populations. Blood examination for circulating microfilariae remains the most diffuse test for *D. repens* diagnosis. However, the Knott’s test, which allows the visualization and the identification of microfilariae, is not familiar to veterinarians in areas of recent introduction of the parasite. Furthermore, an interaction between the two species of *Dirofilaria* has been suggested [33], which seems to slow the spread of *D. immitis* in areas where *D. repens* has firstly settled.

Another aspect which deserves attention is the higher prevalence of human infection by *D. repens* compared to *D. immitis* in Europe, even in countries where the latter is endemic [4]; this is in contrast to the prevalence in the New World, where the human cases of dirofilariosis by *D. immitis* are relatively frequent [293]. There is currently no evidence of a higher virulence of *D. repens* respect to *D. immitis* and of a difference of virulence among strains of the same species, or of a difference in the mosquito vectors of the two parasites. It has been hypothesized that the localization in the subcutaneous tissues may help *D. repens* to escape the natural immune response of unusual hosts, such as humans.

## Conclusions

There is evidence that *D. repens* has spread faster than *D. immitis* from the endemic areas of southern Europe to northern Europe. Climate change affecting mosquito vectors and the facilitation of pet travel seem to have contributed to this expansion; however, the major factor is likely the rate of undiagnosed dogs perpetuating the life-cycle of *D. repens*. Many infected dogs remain undetected due to the subclinical nature of the disease, the lack of rapid and reliable diagnostic tools and the poor knowledge and still low awareness of *D. repens* in non-endemic areas. Research and education should fill this gap. Indeed, improved diagnostic tools are warranted to bring *D. repens* diagnosis to the state of *D. immitis* diagnosis, as well as improved screening of imported dogs and promotion of preventative measures among veterinarians and dog owners. In this respect, to transform the disease in a notifiable disease, at least in humans, would help Europe to have official and comparable data on the presence and variations of prevalence among countries. Upcoming studies should also focus on (i) the vector competence and vectorial capacity of mosquito species; (ii) the presence of different genospecies or genotypes of *D. repens* and their specific interactions with hosts and vectors; and (iii) the possible selection of resistance to macrocyclic lactones if preventative measures increase. For vector-borne diseases where an animal species serves as a reservoir, especially a pet, veterinarians play a significant role in prevention and should be more aware of their responsibility in reducing the impact of the zoonotic agents. In addition, they should enhance multisectorial collaboration with medical entomologists and the public health experts, under the concept (and the actions) of One Health-One Medicine.

## Abbreviations

FYROM: Former Yugoslav Republic of Macedonia
L2: Second larval stage
L3: Third larval stage
PCR: Polymerase chain reaction
